# The genome sequence of *Euphorbia lathyris* L., 1753 (Malpighiales: Euphorbiaceae)

**DOI:** 10.12688/wellcomeopenres.26647.1

**Published:** 2026-05-29

**Authors:** Maarten J. M. Christenhusz, Ilia J. Leitch, Michael F. Fay, Royal Botanic Gardens Kew Genome Acquisition Lab, Plant Genome Sizing Collective, Darwin Tree of Life Barcoding collective, Wellcome Sanger Institute Tree of Life Management, Samples and Laboratory team, Wellcome Sanger Institute Scientific Operations: Sequencing Operations, Wellcome Sanger Institute Tree of Life Core Informatics team, Tree of Life Core Informatics collective, Darwin Tree of Life Consortium

**Affiliations:** 1 Royal Botanic Gardens Kew, Richmond, England, UK; 2 Botanical Society of Britain and Ireland, St Albans, Hertfordshire, England, UK

**Keywords:** Euphorbia lathyris, caper spurge, genome sequence, chromosomal, Malpighiales

## Abstract

We present a genome assembly of *Euphorbia lathyris* (caper spurge; Streptophyta; Magnoliopsida; Malpighiales; Euphorbiaceae). The genome sequence has a total length of 1 020.39 megabases. Most of the assembly (99.88%) is scaffolded into 10 chromosomal pseudomolecules. The mitochondrial sequences have lengths of 886.36, 41.02, 86.31 and 32.73 kilobases and the plastid genome assembly has a length of 163.75 kilobases. Gene annotation of this assembly on Ensembl identified 26 361 protein-coding genes. This assembly was generated as part of the Darwin Tree of Life project, which produces reference genomes for eukaryotic species found in Britain and Ireland.

## Species taxonomy

Eukaryota; Viridiplantae; Streptophyta; Magnoliopsida; Malpighiales; Euphorbiaceae; *Euphorbia*; *Euphorbia lathyris* L., 1753 (NCBI:txid212925).

## Background

Caper spurge is a biennial herb in the Euphorbiaceae. In the first year it grows a plant with a single stem and alternate, linear leaves in four ranks. In the second year these plants branch and bear broadly ovate, opposite leaves below a cyathium: an inflorescence shaped like a cup with four glands, five male flowers and a single female flower. The glands are bilobed, with the lobes tinged purple. The flowers lack petals and the male is formed of just stamens and the female of only a single trilocular ovary. After pollination, the ovary swells to around 1.7 cm in diameter and when ripe, explodes, dispersing the three large seeds.

It is a species from agricultural fields, ruderal sites, gardens and allotments. It is native to Central Asia, but is widely naturalised most temperate parts of the world ( POWO, 2024). In Britain, it is naturalised in shady places in southern England, and it is a frequent casual on waste ground and in gardens elsewhere ( Ministry of Agriculture, Fisheries and Food, 1984; Stace, 2019).

The name *Euphorbia* means ‘good herb’, but Linnaeus (1753) named the genus in honour of the Nubian King and physician Euphorbus ( Christenhusz *et al.*, 2017). Linnaes originally spelled the name as *Euphorbia lathyrus*, but later corrected this ( Linnaeus, 1759, 1762), because he intended to use the Greek *lathyris*, which translates as ‘caper spurge’, rather than the Latin *lathyrus*, which is a pulse (genus *Lathyrus*). Therefore, *Euphorbia lathyris* is the correct spelling ( IPNI, 2024).

Like all *Euphorbia* species, caper spurge contains various toxic compounds, including resins, alkaloids and glycosides ( Ministry of Agriculture, Fisheries and Food, 1984). Studies of medicinal compounds have shown that *E. lathyris* contains anti-inflammatory and cytotoxic substances ( Teng *et al.*, 2018; Wang *et al.*, 2021). The fruits have been the cause of human poisoning when they are mistaken for true capers ( *Capparis spinosa*). *Euphorbia lathyris* has been used to treat a variety of ailments, but it is not commonly used in the UK. As a purgative, its effects are drastic, and it is now rarely used ( Ministry of Agriculture, Fisheries and Food, 1984). Seeds are high in oils and have therefore once been considered a potential crop for liquid fuel production, although the application appears to be limited ( Kingsolver, 1982).

The reported karyotype for *E. lathyrus* is 2 *n* = 20 (e.g. Dempsey *et al.*, 1994), and it appears to be consistent across its range.

We present a chromosome-level genome sequence for *Euphorbia lathyris.* Another assembly (contig-level) is also available for this species (GCA_041381075.1), submitted by the Institute of Botany, Jiangsu Province and Chinese Academy of Sciences (NCBI datasets, O’Leary *et al.*, 2024).

## Methods

### Sample acquisition, flow cytometry and DNA barcoding

**Figure 1.**
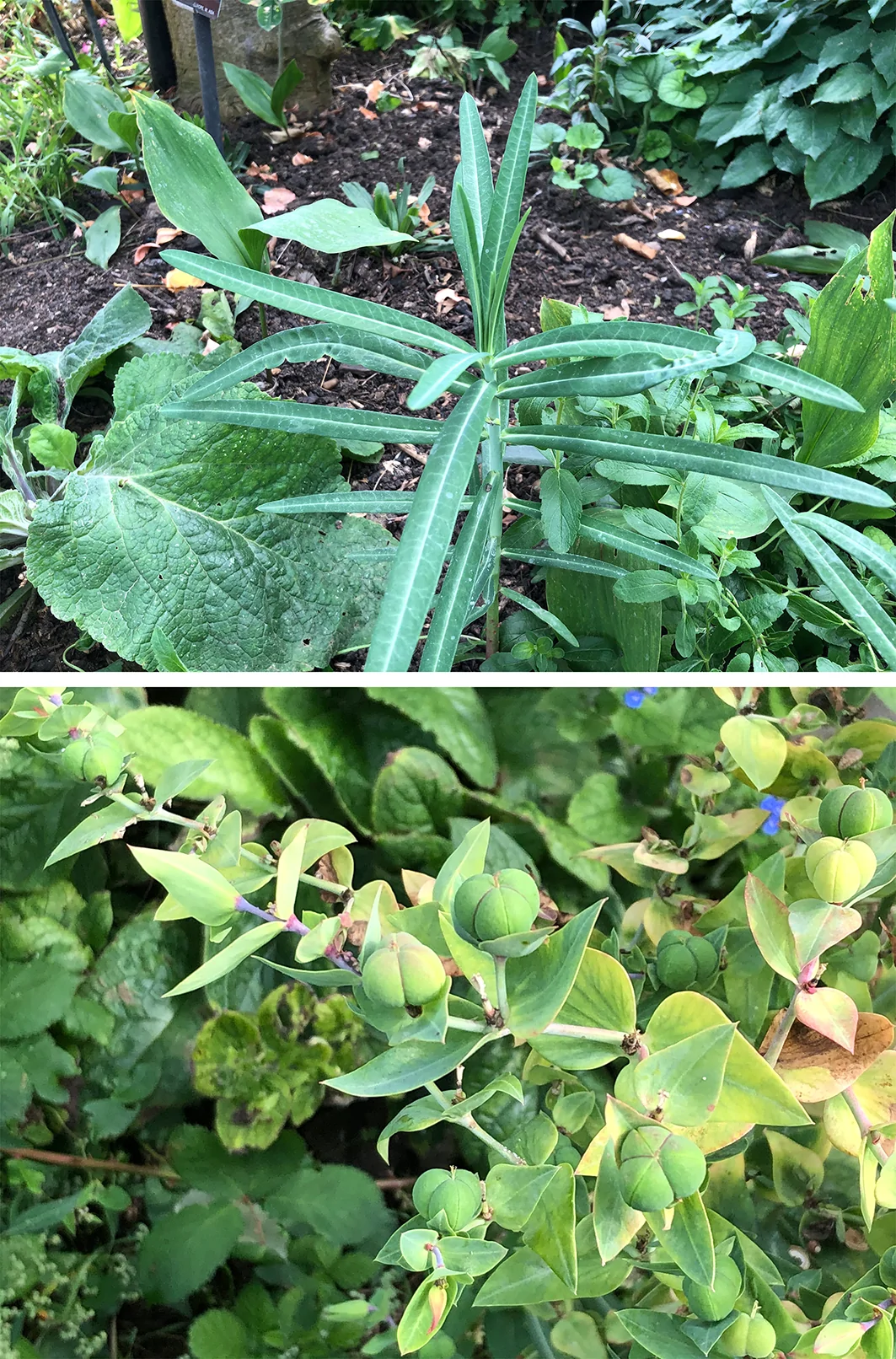
Photographs of the *Euphorbia lathyris* (ddEupLath1) specimen which was sampled for genome sequencing.

A specimen of *Euphorbia lathyris* (specimen ID KDTOL10006, ToLID ddEupLath1; Figure 1) was used for genome sequencing. It was collected from Queen’s Garden, Royal Botanic Gardens Kew, Surrey, UK (latitude 51.4841, longitude −0.2957) on 2020-08-04. The specimen was collected and identified by Maarten J. M. Christenhusz. The same specimen was used for RNA sequencing. The herbarium voucher associated with the sequenced plant is is deposited in the herbarium of RBG Kew (K) (M. Christenhusz 9003).

The genome size was estimated by flow cytometry following the ‘one-step’ method outlined in Pellicer *et al*. (2021) and using propidium iodide as the fluorochrome. The General Purpose Buffer (GPB) supplemented with 3% PVP and 0.08% (v/v) beta-mercaptoethanol was used for isolation of nuclei ( Loureiro *et al.*, 2007), and the internal calibration standard was *Petroselinum crispum* ‘Champion Moss Curled’ with an assumed 1C-value of 2 200 Mb ( Obermayer *et al.*, 2002).

The initial identification was verified by an additional DNA barcoding process according to the framework developed by Twyford *et al*. (2024). Part of the plant specimen was preserved in silica gel desiccant ( Chase & Hills, 1991). DNA extracted from the dried plant was amplified by PCR for standard barcode markers, with the amplicons sequenced and compared to public sequence databases including GenBank and the Barcode of Life Database (BOLD) ( Ratnasingham & Hebert, 2007). Following whole genome sequence generation, the relevant DNA barcode region was also used alongside the initial barcoding data for sample tracking at the WSI ( Twyford *et al.*, 2024). The standard operating procedures for Darwin Tree of Life barcoding are available on protocols.io.

### Nucleic acid extraction

Protocols for high molecular weight (HMW) DNA extraction developed at the Wellcome Sanger Institute (WSI) Tree of Life Core Laboratory are available on protocols.io ( Howard *et al.*, 2025). The ddEupLath1 sample was weighed and triaged to determine the appropriate extraction protocol. Tissue from the leaf was homogenised by cryogenic disruption using the Covaris cryoPREP® Automated Dry Pulverizer. HMW DNA was extracted using the Automated Plant MagAttract v2 protocol. DNA was sheared into an average fragment size of 12–20 kb following the Megaruptor®3 for LI PacBio protocol. Sheared DNA was purified by automated SPRI (solid-phase reversible immobilisation), using AMPure PB beads (Pacific Biosciences) and the Thermo Fisher KingFisher™ Apex to eliminate shorter fragments and concentrate the DNA. The concentration of the sheared and purified DNA was assessed using a Nanodrop spectrophotometer and Qubit Fluorometer using the Qubit dsDNA High Sensitivity Assay kit. Fragment size distribution was evaluated by running the sample on the FemtoPulse system.

RNA was extracted from leaf tissue of ddEupLath1 in the Tree of Life Laboratory at the WSI using the RNA Extraction: Automated MagMax™ *mir*Vana protocol. The RNA concentration was assessed using a Nanodrop spectrophotometer and a Qubit Fluorometer using the Qubit RNA Broad-Range Assay kit. Analysis of the integrity of the RNA was done using the Agilent RNA 6000 Pico Kit and Eukaryotic Total RNA assay.

### PacBio HiFi library preparation and sequencing

Library preparation and sequencing were performed at the WSI Scientific Operations core. Libraries were prepared using the SMRTbell Prep Kit 3.0 (Pacific Biosciences) according to the manufacturer’s instructions. The kit includes reagents for end repair/A-tailing, adapter ligation, post-ligation SMRTbell bead clean-up, and nuclease treatment. Size selection and clean-up were performed using diluted AMPure PB beads (Pacific Biosciences). DNA concentration was quantified using a Qubit Fluorometer v4.0 (ThermoFisher Scientific) and the Qubit 1X dsDNA HS assay kit. Final library fragment size was assessed with the Agilent Femto Pulse Automated Pulsed Field CE Instrument (Agilent Technologies) using the gDNA 55 kb BAC analysis kit.

The sample was sequenced using the Sequel IIe system (Pacific Biosciences, California, USA). The concentration of the library loaded onto the Sequel IIe was in the range 40–135 pM. The SMRT link software, a PacBio web-based end-to-end workflow manager, was used to set-up and monitor the run, and to perform primary and secondary analysis of the data upon completion.

### Hi-C

***Sample preparation and crosslinking***

Hi-C data were generated from the leaf tissue of ddEupLath1 using the Arima-HiC v2 kit (Arima Genomics). Tissue was finely ground using the Covaris cryoPREP Dry Pulverizer (Covaris), and then subjected to nuclei isolation. Nuclei were isolated using a modified protocol based on the Qiagen QProteome Cell Compartment Kit (Qiagen), in which only the Lysis and CE2 buffers were used, with QIAshredder spin columns. After isolation, nuclei were fixed using formaldehyde to a final concentration of 2% to crosslink the DNA. The crosslinked DNA was then digested and biotinylated according to the manufacturer’s instructions. A clean-up step was performed with SPRIselect beads before library preparation. DNA concentration was quantified using the Qubit Fluorometer v4.0 (Thermo Fisher Scientific) and the Qubit HS Assay Kit, following the manufacturer’s instructions.

***Hi-C library preparation and sequencing***

Biotinylated DNA constructs were fragmented using a Covaris E220 sonicator and size selected to 400–600 bp using SPRISelect beads. DNA was enriched with Arima-HiC v2 kit Enrichment beads. End repair, A-tailing, and adapter ligation were carried out with the NEBNext Ultra II DNA Library Prep Kit (New England Biolabs), following a modified protocol where library preparation occurs while DNA remains bound to the Enrichment beads. Library amplification was performed using KAPA HiFi HotStart mix and a custom Unique Dual Index (UDI) barcode set (Integrated DNA Technologies). Depending on sample concentration and biotinylation percentage determined at the crosslinking stage, libraries were amplified with 10–16 PCR cycles. Post-PCR clean-up was performed with SPRISelect beads. Libraries were quantified using the AccuClear Ultra High Sensitivity dsDNA Standards Assay Kit (Biotium) and a FLUOstar Omega plate reader (BMG Labtech).

Prior to sequencing, libraries were normalised to 10 ng/μL. Normalised libraries were quantified again to create equimolar and/or weighted 2.8 nM pools. Pool concentrations were checked using the Agilent 4200 TapeStation (Agilent) with High Sensitivity D500 reagents before sequencing. Sequencing was performed using paired-end 150 bp reads on the Illumina NovaSeq 6000.

### RNA library preparation and sequencing

Libraries were prepared using the NEBNext® Ultra™ II Directional RNA Library Prep Kit for Illumina (New England Biolabs), following the manufacturer’s instructions. Poly(A) mRNA in the total RNA solution was isolated using oligo (dT) beads, converted to cDNA, and uniquely indexed; 14 PCR cycles were performed. Libraries were size-selected to produce fragments between 100–300 bp. Libraries were quantified, normalised, pooled to a final concentration of 2.8 nM, and diluted to 150 pM for loading. Sequencing was carried out on the Illumina NovaSeq 6000 to generate 150-bp paired-end reads.

### Genome assembly

Prior to assembly of the PacBio HiFi reads, a database of *k*-mer counts ( *k* = 31) was generated from the filtered reads using FastK. GenomeScope2 ( Ranallo-Benavidez *et al.*, 2020) was used to analyse the *k*-mer frequency distributions, providing estimates of genome size, heterozygosity, and repeat content.

The HiFi reads were assembled using Hifiasm ( Cheng *et al.*, 2021) with the --primary option. Haplotypic duplications were identified and removed using purge_dups ( Guan *et al.*, 2020). The Hi-C reads ( Rao *et al.*, 2014) were mapped to the primary contigs using bwa-mem2 ( Vasimuddin *et al.*, 2019), and the contigs were scaffolded in YaHS ( Zhou *et al.*, 2023) with the --break option for handling potential misassemblies. The scaffolded assemblies were evaluated using Gfastats ( Formenti *et al.*, 2022), BUSCO ( Manni *et al.*, 2021) and MerquryFK ( Rhie *et al.*, 2020).

The organelle genomes were assembled using OATK ( Zhou *et al.*, 2025).

### Assembly curation

The assembly was decontaminated using the Assembly Screen for Cobionts and Contaminants ( ASCC) pipeline. TreeVal was used to generate the flat files and maps for use in curation. Manual curation was conducted primarily in PretextView and HiGlass ( Kerpedjiev *et al.*, 2018). Scaffolds were visually inspected and corrected as described by Howe *et al*. (2021). Manual corrections included two joins, increasing the scaffold count by 8.6%. The curation process is described at https://gitlab.com/wtsi-grit/rapid-curation . PretextSnapshot was used to generate a Hi-C contact map of the final assembly.

### Assembly quality assessment

The MerquryFK tool ( Rhie *et al.*, 2020) was run in a Singularity container ( Kurtzer *et al.*, 2017) to evaluate *k*-mer completeness and assembly quality for the primary and alternate haplotypes using the *k*-mer database ( *k* = 31) computed prior to genome assembly. The analysis outputs included assembly QV scores and completeness statistics.

The genome was analysed using the BlobToolKit pipeline, a Nextflow implementation of the earlier Snakemake version ( Challis *et al.*, 2020). The pipeline aligns PacBio reads using minimap2 ( Li, 2018) and SAMtools ( Danecek *et al.*, 2021) to generate coverage tracks. It runs BUSCO ( Manni *et al.*, 2021) using lineages identified by querying NCBI datasets ( O’Leary *et al.*, 2024). For the three domain-level lineages, BUSCO genes are aligned to the UniProt Reference Proteomes database ( Bateman *et al.*, 2023) using DIAMOND blastp ( Buchfink *et al.*, 2021). The genome is divided into chunks based on the density of BUSCO genes from the closest taxonomic lineage, and each chunk is aligned to the UniProt Reference Proteomes database with DIAMOND blastx. Sequences without hits are chunked using seqtk and aligned to the NT database with blastn ( Altschul *et al.*, 1990). The BlobToolKit suite consolidates all outputs into a blobdir for visualisation. The BlobToolKit pipeline was developed using nf-core tooling ( Ewels *et al.*, 2020) and MultiQC ( Ewels *et al.*, 2016), with containerisation through Docker ( Merkel, 2014) and Singularity ( Kurtzer *et al.*, 2017).

## Genome sequence report

### Sequence data

**Figure 2.**
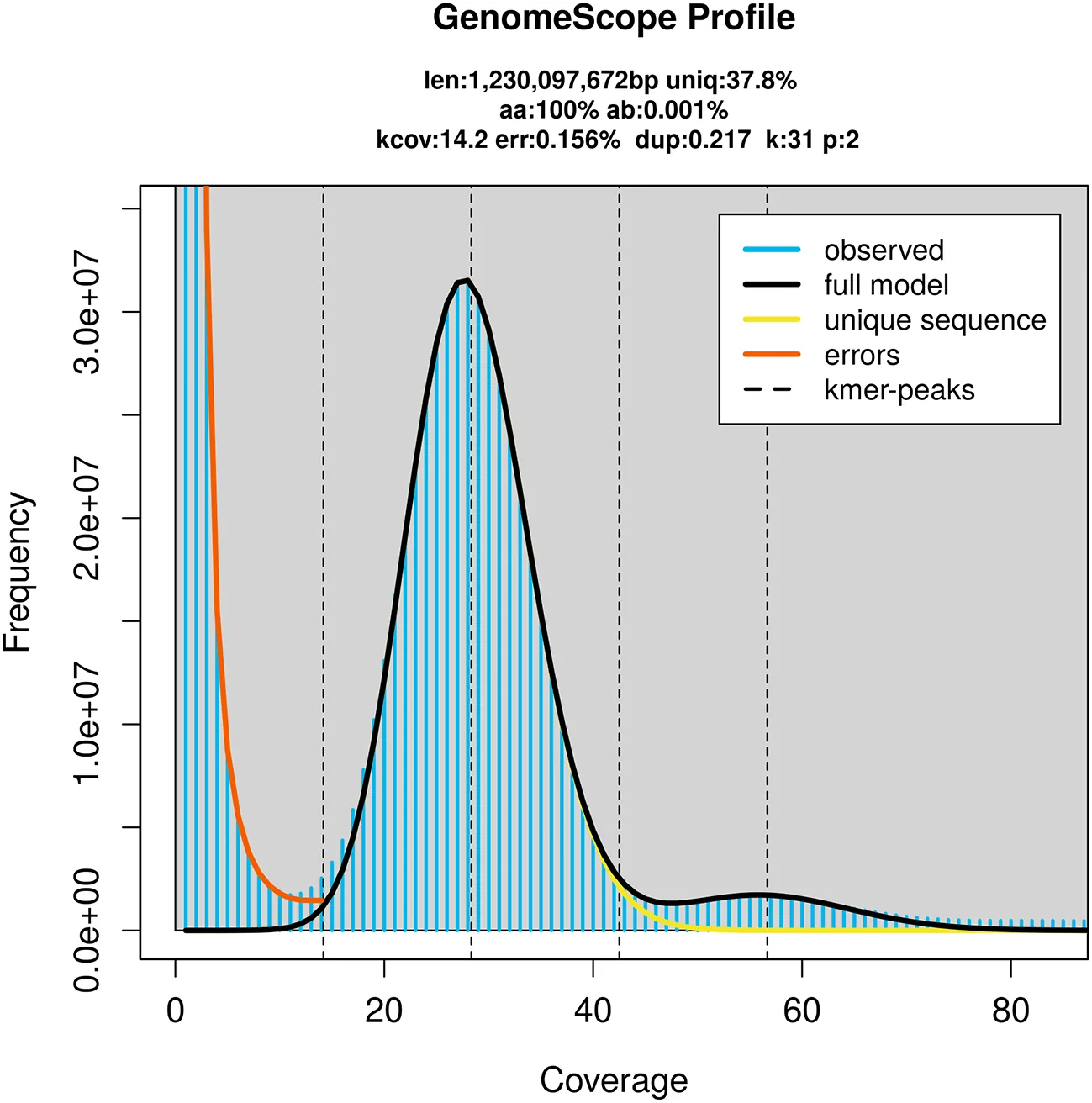
Frequency distribution of *k*-mers generated using GenomeScope2. The plot shows observed and modelled *k*-mer spectra, providing estimates of genome size, heterozygosity, and repeat content based on unassembled sequencing reads.

The genome of a specimen of *Euphorbia lathyris* was sequenced using Pacific Biosciences single-molecule HiFi long reads, generating 36.67 Gb (gigabases) from 2.79 million reads, which were used to assemble the genome. GenomeScope2.0 analysis estimated the haploid genome size at 1 237.60 Mb, with a heterozygosity of 0.06% and repeat content of 61.83% ( Figure 2). Using flow cytometry, the genome size (1C-value) of the sample was estimated to be 1.26 pg, equivalent to 1 230.00 Mb. These estimates guided expectations for the assembly. Based on the estimated genome size, the sequencing data provided approximately 28× coverage. Hi-C sequencing produced 196.68 Gb from 651.24 million reads, which were used to scaffold the assembly. RNA sequencing data were also generated and are available in public sequence repositories. Table 1 summarises the specimen and sequencing details.

**Table 1.** Specimen and sequencing data for *Euphorbia lathyris* (BioProject PRJEB65676).

Platform	PacBio HiFi	Hi-C	RNA-seq
**ToLID**	ddEupLath1	ddEupLath1	ddEupLath1
**Specimen ID**	KDTOL10006	KDTOL10006	KDTOL10006
**BioSample (source individual)**	SAMEA7521715	SAMEA7521715	SAMEA7521715
**BioSample (tissue)**	SAMEA7521810	SAMEA7521813	SAMEA7521813
**Tissue**	leaf	leaf	leaf
**Instrument**	Sequel IIe	Illumina NovaSeq 6000	Illumina NovaSeq 6000
**Run accessions**	ERR12015737; ERR12015738	ERR12035259; ERR12035260	ERR12035257; ERR12035258
**Read count total**	2.79 million	651.24 million	64.77 million
**Base count total**	36.67 Gb	196.68 Gb	19.56 Gb

### Assembly statistics

The primary haplotype was assembled, and contigs corresponding to an alternate haplotype were also deposited in INSDC databases. The final assembly has a total length of 1 020.39 Mb in 33 scaffolds, with 24 gaps, and a scaffold N50 of 96.32 Mb ( Table 2).

**Table 2.** Genome assembly data for *Euphorbia lathyris.*

Genome assembly	Primary assembly
**Assembly name**	ddEupLath1.1
**Assembly accession**	GCA_963576675.1
**Alternate haplotype accession**	GCA_963576625.1
**Assembly level**	chromosome
**Span (Mb)**	1 020.39
**Number of chromosomes**	10
**Number of contigs**	57
**Contig N50**	67.7 Mb
**Number of scaffolds**	33
**Scaffold N50**	96.32 Mb
**Organelles**	Mitochondrial genomes: 886.36; 41.02; 86.31; 32.73 kb; Plastid genome: 163.75 kb

**Figure 3.**
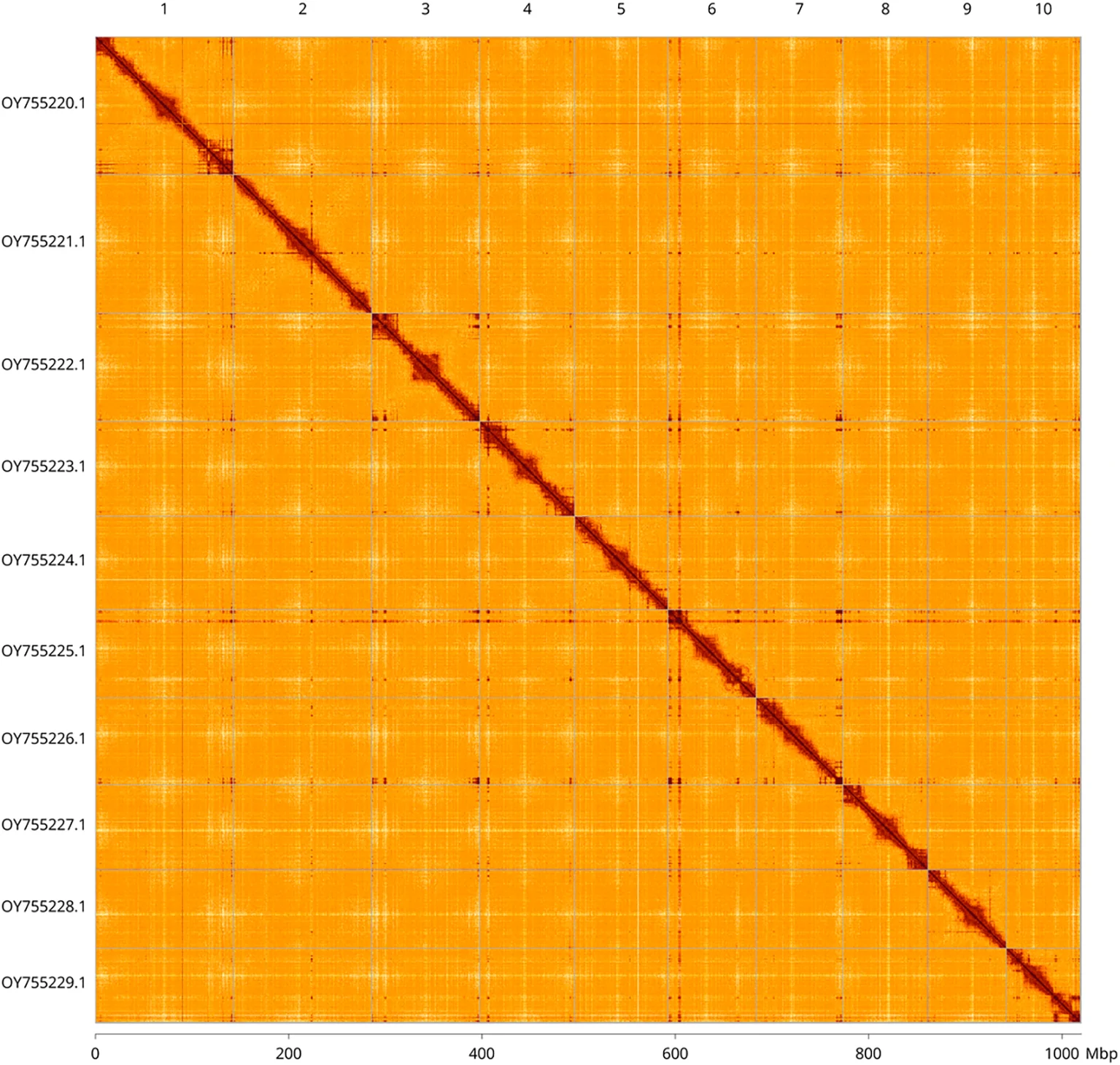
Hi-C contact map of the *euphorbia lathyris* genome assembly. Assembled chromosomes are shown in order of size and labelled along the axes, with a megabase scale shown below. The plot was generated using PretextSnapshot.

Most of the assembly sequence (99.88%) was assigned to 10 chromosomal-level scaffolds. These chromosome-level scaffolds, confirmed by Hi-C data, are named according to size ( Figure 3; Table 3).

**Table 3.** Chromosomal pseudomolecules in the primary genome assembly of *Euphorbia lathyris* ddEupLath1.

INSDC accession	Molecule	Length (Mb)	GC%
OY755220.1	1	143.14	37
OY755221.1	2	142.89	37
OY755222.1	3	111.91	37
OY755223.1	4	98.13	37
OY755224.1	5	96.32	37
OY755225.1	6	91.35	37
OY755226.1	7	89.76	37
OY755227.1	8	88.06	37
OY755228.1	9	81.19	37
OY755229.1	10	76.46	36.50

Multiple mitochondrial genomes (four) and the plastid genome (length 163.75 kb, OY755234.1) were also assembled. These sequences are included as contigs in the multifasta file of the genome submission and as standalone records.

### Assembly quality metrics

**Figure 4.**
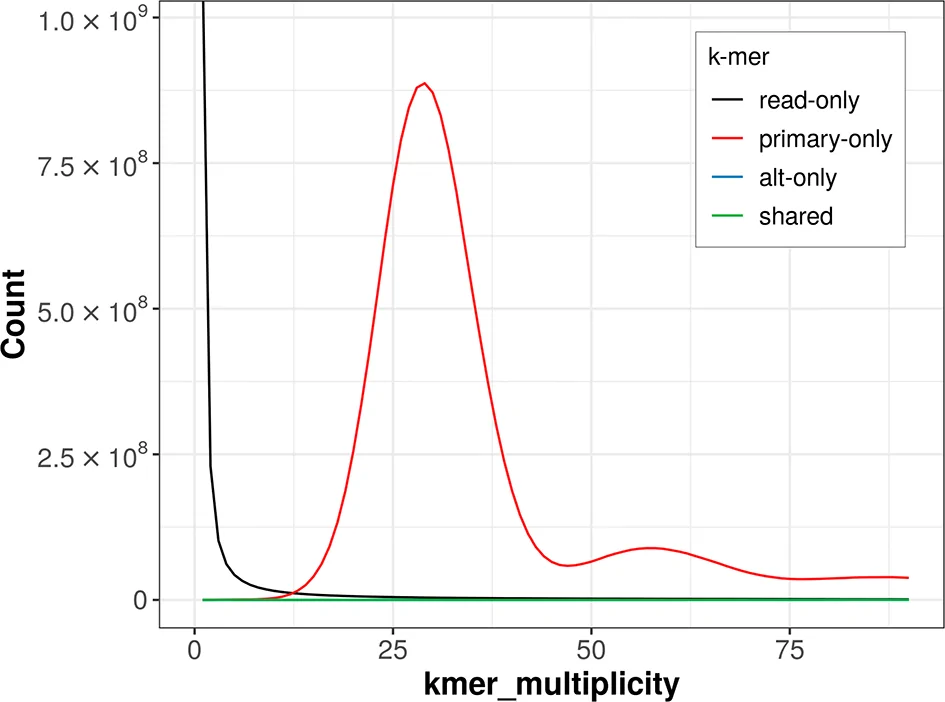
Evaluation of *k*-mer completeness using MerquryFK. This plot illustrates the recovery of *k*-mers from the original read data in the final assemblies. The horizontal axis represents *k*-mer multiplicity, and the vertical axis shows the number of *k*-mers. The black curve represents *k*-mers that appear in the reads but are not assembled. The green curve corresponds to *k*-mers shared by both haplotypes, and the red and blue curves show *k*-mers found only in one of the haplotypes.

The combined primary and alternate assemblies achieve an estimated QV of 69.5. The *k*-mer completeness is 98.10% for the primary assembly, 0.11% for the alternate haplotype, and 98.12% for the combined assemblies ( Figure 4).

**Figure 5.**
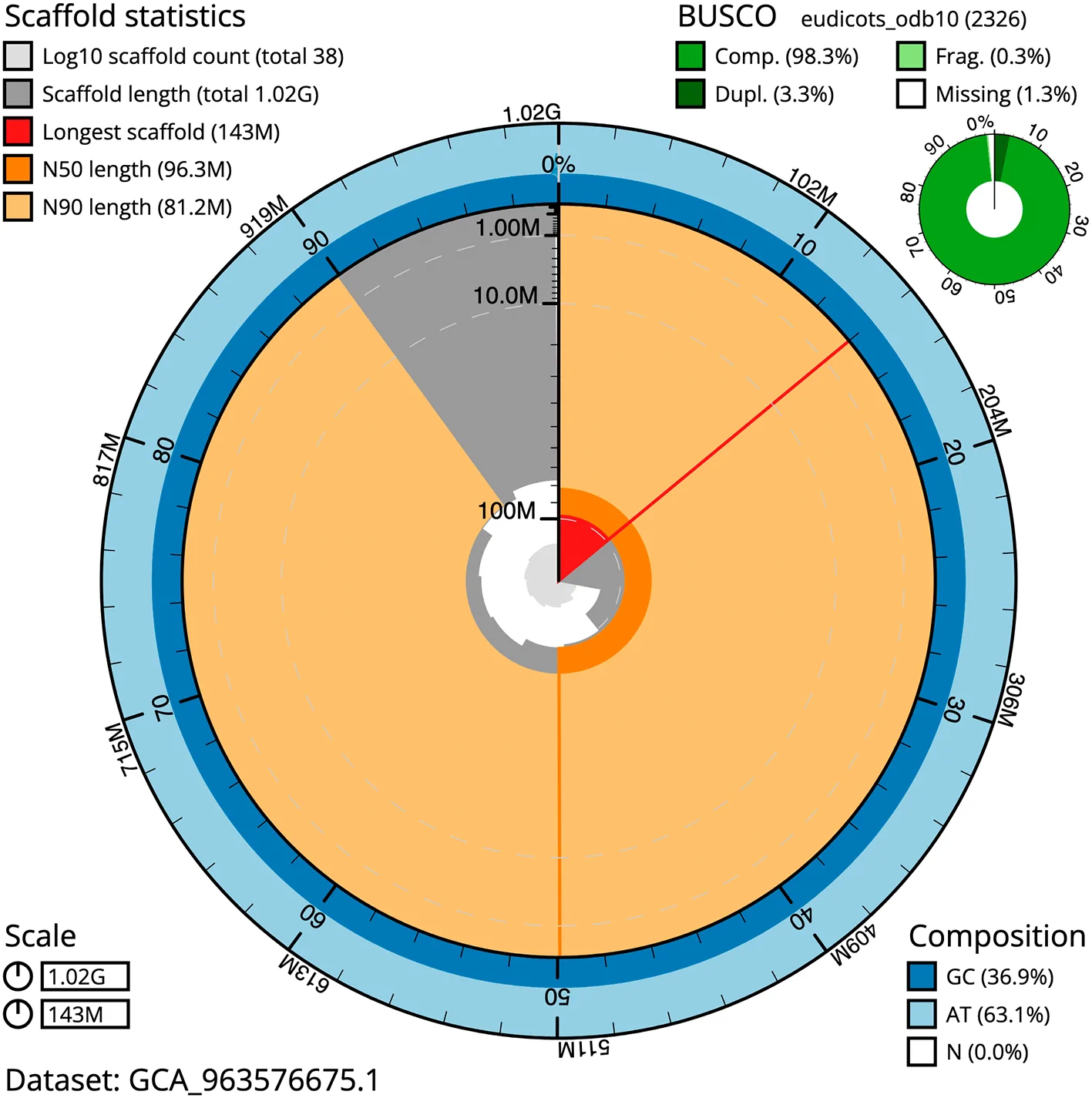
Assembly metrics for ddEupLath1.1. The BlobToolKit snail plot provides an overview of assembly metrics and BUSCO gene completeness. The circumference represents the length of the whole genome sequence, and the main plot is divided into 1,000 bins around the circumference. The outermost blue tracks display the distribution of GC, AT, and N percentages across the bins. Scaffolds are arranged clockwise from longest to shortest and are depicted in dark grey. The longest scaffold is indicated by the red arc, and the deeper orange and pale orange arcs represent the N50 and N90 lengths. A light grey spiral at the centre shows the cumulative scaffold count on a logarithmic scale. A summary of complete, fragmented, duplicated, and missing BUSCO genes in the set is presented at the top right. An interactive version of this figure can be accessed on the BlobToolKit viewer.

**Figure 6.**
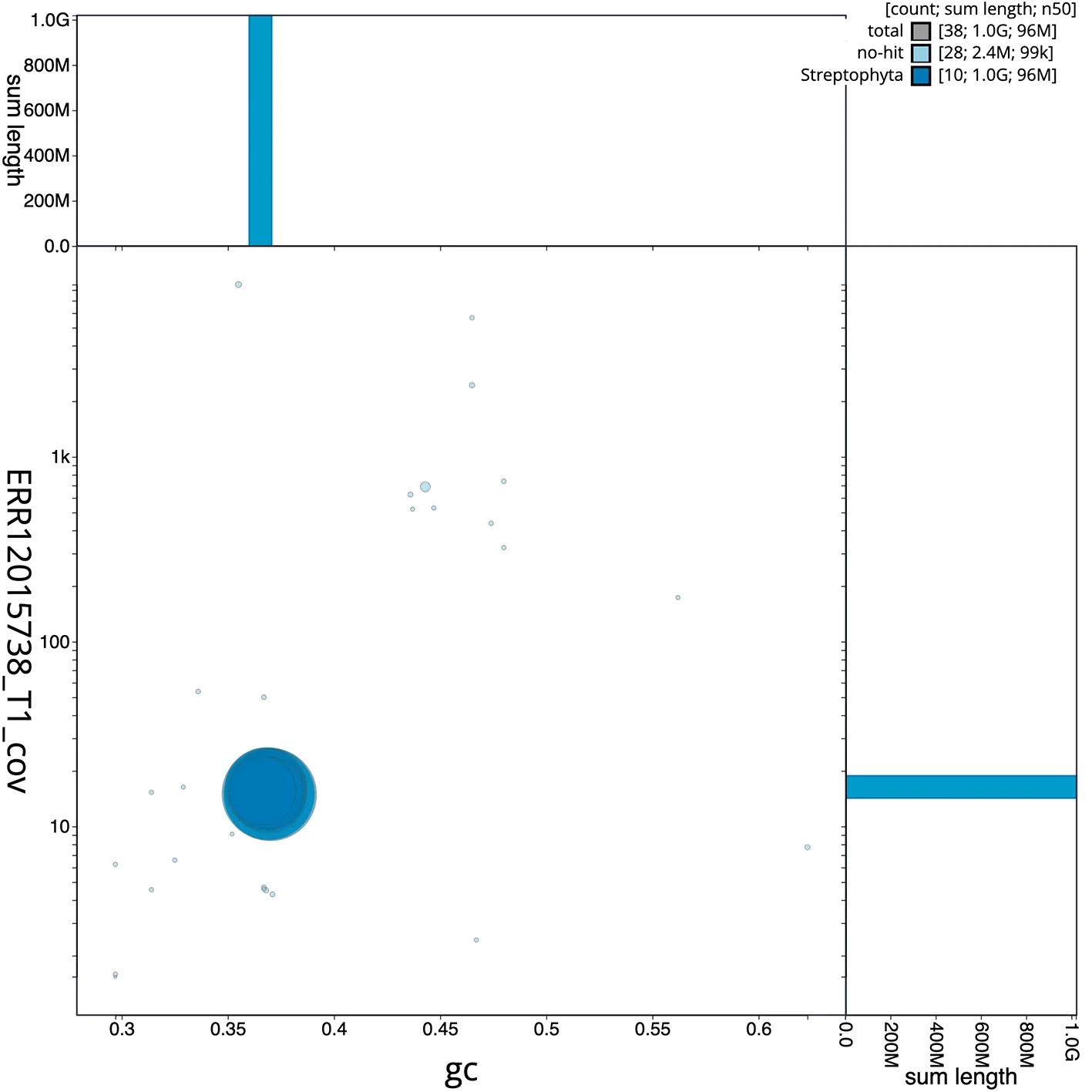
BlobToolKit blob plot for ddEupLath1.1. The plot shows base coverage (vertical axis) and GC content (horizontal axis). The circles represent scaffolds, with the size proportional to scaffold length and the colour representing phylum membership. The histograms along the axes display the total length of sequences distributed across different levels of coverage and GC content. An interactive version of this figure is available on the BlobToolKit viewer.

BUSCO v.5.5.0 analysis using the eudicots_odb10 dataset ( *n* = 2326) showed 98.3% complete BUSCOs, including 3.3% duplicated BUSCOs, with 0.3% fragmented and 1.3% missing. The snail plot in Figure 5 summarises the scaffold length distribution and other assembly statistics for the primary assembly. The blob plot in Figure 6 shows the distribution of scaffolds by GC proportion and coverage.

Table 4 lists the assembly metric benchmarks adapted from Rhie *et al.* (2021) and the Earth BioGenome Project Report on Assembly Standards September 2024. The EBP metric calculated for the primary assembly is **7.C.Q69**, meeting the recommended reference standard.

**Table 4.** Earth Biogenome Project summary metrics for the *Euphorbia lathyris* assembly.

Measure	Value	Benchmark
EBP summary (primary)	7.C.Q69	6.C.Q40
Contig N50 length	67.70 Mb	≥ 1 Mb
Scaffold N50 length	96.32 Mb	= chromosome N50
Consensus quality (QV)	Primary: 69.9; alternate: 53.7; combined: 69.5	≥ 40
*k*-mer completeness	Primary: 98.10%; alternate: 0.11%; combined: 98.12%	≥ 95%
BUSCO	C:98.3%[D:3.3%],F:0.3%,M:1.3%,n:2326	S > 90%; D < 5%
Percentage of assembly assigned to chromosomes	99.88%	≥ 90%

**Notes:** The EBP summary uses log10(Contig N50); chromosome-level (C) or log10(Scaffold N50); Q (Merqury QV). BUSCO: C = complete; S = single-copy; D = duplicated; F = fragmented; M = missing; n = orthologues

**Table 5.** Software versions and sources used for *euphorbia lathyris.*

Software	Version	Source
BLAST	2.14.0	ftp://ftp.ncbi.nlm.nih.gov/blast/executables/blast+/
BlobToolKit	4.3.3	https://github.com/blobtoolkit/blobtoolkit
BUSCO	5.5.0	https://gitlab.com/ezlab/busco
bwa-mem2	2.2.1	https://github.com/bwa-mem2/bwa-mem2
DIAMOND	2.1.8	https://github.com/bbuchfink/diamond
fasta_windows	0.2.4	https://github.com/tolkit/fasta_windows
FastK	1.1	https://github.com/thegenemyers/FASTK
GenomeScope2.0	2.0.1	https://github.com/tbenavi1/genomescope2.0
Gfastats	1.3.6	https://github.com/vgl-hub/gfastats
Hifiasm	0.19.5-r587	https://github.com/chhylp123/hifiasm
HiGlass	1.13.4	https://github.com/higlass/higlass
MerquryFK	1.1.2	https://github.com/thegenemyers/MERQURY.FK
Minimap2	2.24-r1122	https://github.com/lh3/minimap2
Oatk	0.9	https://github.com/c-zhou/oatk
MultiQC	1.14; 1.17 and 1.18	https://github.com/MultiQC/MultiQC
Nextflow	23.04.1	https://github.com/nextflow-io/nextflow
PretextSnapshot	0.0.5	https://github.com/sanger-tol/PretextSnapshot
PretextView	1.0.3	https://github.com/sanger-tol/PretextView
purge_dups	1.2.3	https://github.com/dfguan/purge_dups
samtools	1.18	https://github.com/samtools/samtools
sanger-tol/ascc	0.1.0	https://github.com/sanger-tol/ascc
sanger-tol/blobtoolkit	0.3.0	https://github.com/sanger-tol/blobtoolkit
sanger-tol/curationpretext	1.4.2	https://github.com/sanger-tol/curationpretext
Seqtk	1.3	https://github.com/lh3/seqtk
Singularity	3.9.0	https://github.com/sylabs/singularity
TreeVal	1.4.0	https://github.com/sanger-tol/treeval
YaHS	1.1a.2	https://github.com/c-zhou/yahs

### Genome annotation report

The *Euphorbia lathyris* genome assembly (GCA_963576675.1) was annotated by Ensembl at the European Bioinformatics Institute (EBI). This annotation includes 56 393 transcribed mRNAs from 26 361 protein-coding and 14 306 non-coding genes. The average transcript length is 3 455.55 bp, with an average of 1.39 coding transcripts per gene and 4.57 exons per transcript. For further information about the annotation, please refer to the annotation page on Ensembl.

## Author information

• Members of the Royal Botanic Gardens Kew Genome Acquisition Lab
• Members of the Plant Genome Sizing Collective
• Members of the Darwin Tree of Life Barcoding collective
• Members of the Wellcome Sanger Institute Tree of Life Management, Samples and Laboratory team
• Members of Wellcome Sanger Institute Scientific Operations – Sequencing Operations
• Members of the Wellcome Sanger Institute Tree of Life Core Informatics team
• Members of the Tree of Life Core Informatics collective
• Members of the Darwin Tree of Life Consortium

## Wellcome sanger institute – Legal and governance

The materials that have contributed to this genome note have been supplied by a Darwin Tree of Life Partner. The submission of materials by a Darwin Tree of Life Partner is subject to the **‘Darwin Tree of Life Project Sampling Code of Practice’**, which can be found in full on the Darwin Tree of Life website. By agreeing with and signing up to the Sampling Code of Practice, the Darwin Tree of Life Partner agrees they will meet the legal and ethical requirements and standards set out within this document in respect of all samples acquired for, and supplied to, the Darwin Tree of Life Project. Further, the Wellcome Sanger Institute employs a process whereby due diligence is carried out proportionate to the nature of the materials themselves, and the circumstances under which they have been/are to be collected and provided for use. The purpose of this is to address and mitigate any potential legal and/or ethical implications of receipt and use of the materials as part of the research project, and to ensure that in doing so we align with best practice wherever possible. The overarching areas of consideration are:

• Ethical review of provenance and sourcing of the material
• Legality of collection, transfer and use (national and international)

Each transfer of samples is further undertaken according to a Research Collaboration Agreement or Material Transfer Agreement entered into by the Darwin Tree of Life Partner, Genome Research Limited (operating as the Wellcome Sanger Institute), and in some circumstances, other Darwin Tree of Life collaborators.

## Data Availability

European Nucleotide Archive: Euphorbia lathyris. Accession number PRJEB65676; BioProject URL: https://identifiers.org/ena.embl/PRJEB65676. The genome sequence is released openly for reuse. The *Euphorbia lathyris* genome sequencing initiative is part of the Darwin Tree of Life Project (PRJEB40665) and the Sanger Institute Tree of Life Programme (PRJEB43745). All raw sequence data and the assembly have been deposited in INSDC databases. Raw data and assembly accession identifiers are reported in Tables 1 and 2. Pipelines used for genome assembly at the WSI Tree of Life are available at https://pipelines.tol.sanger.ac.uk/pipelines. Table 5 lists software versions used in this study.

## References

[ref1] Altschul SF Gish W Miller W : Basic Local Alignment Search Tool. *Journal of Molecular Biology.* 1990;215(3):403–410. 10.1016/S0022-2836(05)80360-2 2231712

[ref2] Bateman A Martin M-J Orchard S : UniProt: The Universal Protein Knowledgebase in 2023. *Nucleic Acids Res.* 2023;51(D1):D523–D531. 10.1093/nar/gkac1052 36408920 PMC9825514

[ref3] Buchfink B Reuter K Drost H-G : Sensitive protein alignments at tree-of-life scale using DIAMOND. *Nat Methods.* 2021;18(4):366–368. 10.1038/s41592-021-01101-x 33828273 PMC8026399

[ref4] Challis R Richards E Rajan J : BlobToolKit – interactive quality assessment of genome assemblies. *G3: Genes, Genomes, Genetics.* 2020;10(4):1361–1374. 10.1534/g3.119.400908 32071071 PMC7144090

[ref5] Chase MW Hills HH : Silica gel: An ideal material for field preservation of leaf samples for DNA studies. *Taxon.* 1991;40(2):215–220. 10.2307/1222975

[ref6] Cheng H Concepcion GT Feng X : Haplotype-resolved *de novo* assembly using phased assembly graphs with Hifiasm. *Nat Methods.* 2021;18(2):170–175. 10.1038/s41592-020-01056-5 33526886 PMC7961889

[ref7] Christenhusz MJM Fay MF Chase MW : *Plants of the World: An Illustrated Encyclopedia of Vascular Plant Families.* Richmond; Chicago: Kew Publishing; University of Chicago Press;2017.

[ref8] Danecek P Bonfield JK Liddle J : Twelve years of SAMtools and BCFtools. *GigaScience.* 2021;10(2):giab008. 10.1093/gigascience/giab008 33590861 PMC7931819

[ref9] Dempsey RE Gornall RJ Bailey JP : Contributions to a cytological catalogue of the British and Irish flora, 4. *Watsonia.* 1994;20:63–66.

[ref10] Ewels P Magnusson M Lundin S : MultiQC: Summarize analysis results for multiple tools and samples in a single report. *Bioinformatics.* 2016;32(19):3047–3048. 10.1093/bioinformatics/btw354 27312411 PMC5039924

[ref11] Ewels PA Peltzer A Fillinger S : The nf-core framework for community-curated bioinformatics pipelines. *Nature Biotechnology.* 2020;38(3):276–278. 10.1038/s41587-020-0439-x 32055031

[ref12] Formenti G Abueg L Brajuka A : Gfastats: Conversion, evaluation and manipulation of genome sequences using assembly graphs. *Bioinformatics.* 2022;38(17):4214–4216. 10.1093/bioinformatics/btac460 35799367 PMC9438950

[ref13] Guan D McCarthy SA Wood J : Identifying and removing haplotypic duplication in primary genome assemblies. *Bioinformatics.* 2020;36(9):2896–2898. 10.1093/bioinformatics/btaa025 31971576 PMC7203741

[ref14] Howard C Denton A Jackson B : On the path to reference genomes for all biodiversity: Lessons learned and laboratory protocols created in the Sanger Tree of Life core laboratory over the first 2000 species. *bioRxiv.* 2025. 10.1101/2025.04.11.648334 PMC12548527 41129326

[ref15] Howe K Chow W Collins J : Significantly improving the quality of genome assemblies through curation. *GigaScience.* 2021;10(1):giaa153. 10.1093/gigascience/giaa153 33420778 PMC7794651

[ref16] IPNI: *Euphorbia lathyris* L. 2024. Reference Source

[ref17] Kerpedjiev P Abdennur N Lekschas F : HiGlass: Web-based visual exploration and analysis of genome interaction maps. *Genome Biol.* 2018;19(1):125. 10.1186/s13059-018-1486-1 30143029 PMC6109259

[ref18] Kingsolver BE : *Euphorbia lathyris* reconsidered: Its potential as an energy crop for arid lands. *Biomass.* 1982;2(4):281–298. 10.1016/0144-4565(82)90014-2

[ref19] Kurtzer GM Sochat V Bauer MW : Singularity: Scientific containers for mobility of compute. *PLoS One.* 2017;12(5):e0177459. 10.1371/journal.pone.0177459 28494014 PMC5426675

[ref20] Li H : Minimap2: Pairwise alignment for nucleotide sequences. *Bioinformatics.* 2018;34(18):3094–3100. 10.1093/bioinformatics/bty191 29750242 PMC6137996

[ref21] Linnaeus C : Species Plantarum. 1753.

[ref22] Linnaeus C : Systema Naturae. 1759;2.

[ref23] Linnaeus C : Species Plantarum. 1762;1.

[ref24] Loureiro J Rodriguez E Doležel J : Two new nuclear isolation buffers for plant DNA flow cytometry: A test with 37 species. *Ann Bot.* 2007;100(4):875–888. 10.1093/aob/mcm152 17684025 PMC2749623

[ref25] Manni M Berkeley MR Seppey M : BUSCO update: Novel and streamlined workflows along with broader and deeper phylogenetic coverage for scoring of eukaryotic, prokaryotic, and viral genomes. *Mol Biol Evol.* 2021;38(10):4647–4654. 10.1093/molbev/msab199 34320186 PMC8476166

[ref26] Merkel D : Docker: Lightweight Linux containers for consistent development and deployment. *Linux J.* 2014;2014(239):2. 10.5555/2600239.2600241

[ref27] Ministry of Agriculture, Fisheries and Food: *Poisonous Plants in Britain and Their Effects on Animals and Man.* London: Her Majesty’s Stationery Office;1984.

[ref28] O’Leary NA Cox E Holmes JB : Exploring and retrieving sequence and metadata for species across the tree of life with NCBI Datasets. *Sci Data.* 2024;11(1):732. 10.1038/s41597-024-03571-y 38969627 PMC11226681

[ref29] Obermayer R Leitch IJ Hanson L : Nuclear DNA C-values in 30 species double the familial representation in pteridophytes. *Ann Bot.* 2002;90(2):209–217. 10.1093/aob/mcf167 12197518 PMC4240412

[ref30] Pellicer J Powell RF Leitch IJ : The application of flow cytometry for estimating genome size, ploidy level endopolyploidy, and reproductive modes in plants. Besse P , editor. *Methods in Molecular Biology.* New York, NY:2021;2222:325–61. 10.1007/978-1-0716-0997-2_17 33301101

[ref31] POWO: Plants of the world online. 2024. Reference Source

[ref32] Ranallo-Benavidez TR Jaron KS Schatz MC : GenomeScope 2.0 and Smudgeplot for reference-free profiling of polyploid genomes. *Nat Commun.* 2020;11(1):1432. 10.1038/s41467-020-14998-3 32188846 PMC7080791

[ref33] Rao SSP Huntley MH Durand NC : A 3D map of the human genome at kilobase resolution reveals principles of chromatin looping. *Cell.* 2014;159(7):1665–1680. 10.1016/j.cell.2014.11.021 25497547 PMC5635824

[ref34] Ratnasingham S Hebert PDN : BOLD: The Barcode of Life Data System (http://www.barcodinglife.org). *Molecular Ecology Notes.* 2007;7(3):355–364. 10.1111/j.1471-8286.2007.01678.x 18784790 PMC1890991

[ref35] Rhie A McCarthy SA Fedrigo O : Towards complete and error-free genome assemblies of all vertebrate species. *Nature.* 2021;592(7856):737–746. 10.1038/s41586-021-03451-0 33911273 PMC8081667

[ref36] Rhie A Walenz BP Koren S : Merqury: Reference-free quality, completeness, and phasing assessment for genome assemblies. *Genome Biol.* 2020;21(1):245. 10.1186/s13059-020-02134-9 32928274 PMC7488777

[ref37] Stace CA : *New Flora of the British Isles.* Middlewood Green: C&M Floristics;2019.

[ref38] Teng Y-N Wang Y Hsu P-L : Mechanism of action of cytotoxic compounds from the seeds of *Euphorbia lathyris.* *Phytomedicine.* 2018;41:62–66. 10.1016/j.phymed.2018.02.001 29519320 PMC6496940

[ref39] Twyford AD Beasley J Barnes I : A DNA barcoding framework for taxonomic verification in the Darwin Tree of Life Project. *Wellcome Open Res.* 2024;9:339. 10.12688/wellcomeopenres.21143.1 39386966 PMC11462125

[ref40] Vasimuddin M Misra S Li H : Efficient architecture-aware acceleration of BWA-MEM for multicore systems. *2019 IEEE International Parallel and Distributed Processing Symposium (IPDPS).* IEEE;2019;314–24. 10.1109/IPDPS.2019.00041

[ref41] Wang Y Song Z Guo Y : Diterpenoids from the seeds of *Euphorbia lathyris* and their anti-inflammatory activity. *Bioorganic Chemistry.* 2021;112:104944. 10.1016/j.bioorg.2021.104944 33962090

[ref42] Zhou C Brown M Blaxter M : Oatk: A de novo assembly tool for complex plant organelle genomes. *Genome Biology.* 2025;26(1):235. 10.1186/s13059-025-03676-6 40775726 PMC12329965

[ref43] Zhou C McCarthy SA Durbin R : YaHS: Yet another Hi-C scaffolding tool. *Bioinformatics.* 2023;39(1):btac808. 10.1093/bioinformatics/btac808 36525368 PMC9848053

