# Supplementary material for: The genome sequence of Euphorbia lathyris L., 1753 (Malpighiales: Euphorbiaceae)
Source: Wellcome Open Res. 2026 May 29;11:322. [Version 1] doi: 10.12688/wellcomeopenres.26647.1 (PMC13550919; doi:10.12688/wellcomeopenres.26647.1)
Supplement: Peer review history

# Reviewer report: Reviewer response for version 1

**DOI:** 10.21956/wellcomeopenres.29344.r158423

**Published:** 2026-07-08

**Authors:** Kishor Gaikwad

**Affiliations:** 1 ICAR-National Institute for Plant Biotechnology, Pusa Campus, New Delhi, India

1 Major Concerns

 1.1 Genome Size Discrepancy (~18% Missing)

 The assembly span (1,020.39 Mb) is substantially smaller than both the GenomeScope2 estimate (1,237.60 Mb) and the flow cytometry estimate (1,230.00 Mb). This ~18% gap is not discussed. The authors should clarify whether this reflects collapsed heterozygous regions, unassembled repetitive DNA, biological variation, or a combination of factors.

 1.2 Alternate Haplotype Quality Is Extremely Low

 The alternate haplotype has a QV of only 53.7 and k-mer completeness of 0.11%. This is far below usable standards and suggests the alternate assembly is largely artifactual. The authors should either explain why the alternate is biologically or technically limited, justify its deposition, or consider omitting it.

 1.3 Mitochondrial Genome Structure Is Unclear

 Four mitochondrial contigs were assembled, including an exceptionally large 886.36 kb contig. The multipartite structure is unusual. The authors should clarify whether this represents a genuine multipartite mitochondrial genome, assembly fragmentation, nuclear mitochondrial DNA segments (numts), or contamination.

 1.4 Manual Curation Description Is Confusing

 The authors state that "two joins" increased the scaffold count by 8.6%. Joins should decrease scaffold count. This needs clarification, were there accompanying breaks, or is this a reporting error? The pre-curation scaffold count should also be provided.

 2. Minor Issues (Grammatical)

- Figure 3 caption: "euphorbia lathyris" should be " *Euphorbia lathyris*" (italicized, capitalized).
- Page 10: "meeting the recommended reference" is an incomplete sentence.
- Figure 5 reports 38 scaffolds, but Table 2 reports 33 - discrepancy needs resolution.
- Main text: "Zhou et al., 20" should be "Zhou et al., 2025" (citation for OATK).

3. Conclusion

 This is a strong genome note with a high-quality chromosome-level assembly that meets EBP reference standards. The four major concerns are readily addressable and do not undermine the core contribution. I recommend Minor Revision contingent on satisfactory responses to the points raised above.

Are sufficient details of methods and materials provided to allow replication by others?

Yes

Is the rationale for creating the dataset(s) clearly described?

Yes

Are the datasets clearly presented in a useable and accessible format?

Yes

Are the protocols appropriate and is the work technically sound?

Yes

Reviewer Expertise:

Plant genomics, focus on legumes

I confirm that I have read this submission and believe that I have an appropriate level of expertise to confirm that it is of an acceptable scientific standard.

# Reviewer report: Reviewer response for version 1

**DOI:** 10.21956/wellcomeopenres.29344.r158424

**Published:** 2026-07-01

**Authors:** Pär K Ingvarsson

**Affiliations:** 1 Swedish University of Agricultural Sciences, Uppsala, Sweden

The manuscript described a chromosome-level genome assembly of *Euphorbia lathyris* L. (caper spurge). Key findings include a genome size of 1.02 Gb of which 99.88% was scaffolded into 10 chromosomal pseudomolecules. The annotation identified 26,361 protein-coding genes. The organelle genomes were assembled into four (mtDNA) or a single (cpDNA) sequence. The sequenced sample was obtained from RBG Kew. Sequencing employed Pacific Biosciences HiFi technology (Illumina NovaSeq 6000 for Hi-C scaffolding) and RNA-seq for annotation support. Assembly used Hifiasm with YaHS scaffolding and MerquryFK validation. The assembly meets Earth BioGenome Project standards with a scaffold N50 of 96.32 Mb, contig N50 of 67.70 Mb and a BUSCO completeness of 98.3%. All data are publicly available under BioProject, supporting future phylogenomic and evolutionary research within the broader Darwin Tree of Life initiative. The methods in the manuscript are well described, and the results are of high quality.

Are sufficient details of methods and materials provided to allow replication by others?

Yes

Is the rationale for creating the dataset(s) clearly described?

Yes

Are the datasets clearly presented in a useable and accessible format?

Yes

Are the protocols appropriate and is the work technically sound?

Yes

Reviewer Expertise:

plant genomics, computational biology, bioinformatics

I confirm that I have read this submission and believe that I have an appropriate level of expertise to confirm that it is of an acceptable scientific standard.
